# Development of an Electrochemical Sensing Technique for Rapid Genotyping of Hepatitis B Virus

**DOI:** 10.3390/s140305611

**Published:** 2014-03-20

**Authors:** Jinyuan Chen, Shaohuang Weng, Qingqiong Chen, Ailin Liu, Fengqing Wang, Jing Chen, Qiang Yi, Qicai Liu, Xinhua Lin

**Affiliations:** 1 Department of Pharmaceutical Analysis, Fujian Medical University, Fuzhou 350005, China; E-Mails:cjyfjmu@163.com (J.C.); shweng@mail.fjmu.edu.cn (S.W.); liuailinlal@163.com (A.L.); 2 Department of Laboratory Medicines, Fujian Medical University, Fuzhou 350004, China; E-Mail: cqq036@126.com; 3 Department of Laboratory Medicine, the First Affiliated Hospital, Fujian Medical University, Fuzhou 35000, China; E-Mails: fengfang77777@aliyun.com (F.W.); chenear9040@sina.com (J.C.); yiqiang1499@163.com (Q.Y.); 4 Department of Laboratory Medicine, Medical Technology and Engineering College, Fujian Medical University, Fuzhou 35000, China

**Keywords:** HBV genotypes, biosensors, RuHex

## Abstract

To develop a convenient; sensitive; accurate; and economical technique for genotyping of hepatitis B viruses (HBVs).

**Methods:**

The mercapto-modified B1; B2; C1; and C2-specific genotyping probes consisted of two probes for each HBV genotype that served as a double verification system. These probes were fixed on the surface of No. 1; 2; 3; and 4 gold electrodes; respectively; via Au-S bonds. Different charge generated by the binding of RuHex to phosphate groups of the DNA backbone before and after hybridization was used for distinguishing the different genotypes.

**Results:**

During hybridization with genotype B; the charges detected at the No. 1 and 2 electrodes were significantly increased; while the charge at the No. 3 and 4 electrodes did not change significantly. During hybridization with genotype C; the charges detected at No. 3 and 4 electrodes were significantly increased; while the signals remained unchanged at the No. 1 and 2 electrodes. During hybridization with mixed genotypes (B and C); the charges detected at all four electrodes were significantly increased. The linear range of detection was 10^−7^ to 10^−10^ mol/L and the sensitivity for detecting mixed B (10%) or C (10%).

**Conclusions:**

Rapid genotyping of HBVs based on electrochemical sensing is simple, has good specificity; and can greatly reduce the cost. This method can be used for sensitive detection of mixed B and C HBV genotypes.

## 1. Introduction

**Figure 1.**
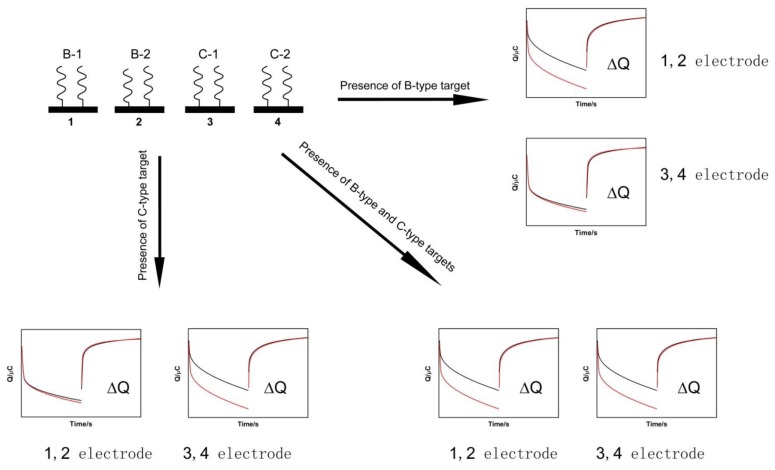
Schematic representation of the chronocoulometric DNA biosensor. B type HBV is determined when the signals of electrodes No. 1 and No. 2 increase simultaneously after hybridization. C type is determined when the signals of electrodes No. 3 and No. 4 increase simultaneously after hybridization. The mixed type is determined when the signals of No. 1, No. 2, No. 3 and No. 4 electrodes increase simultaneously after hybridization. The type cannot be confirmed when the signals of electrodes No. 1 and No. 3 or No. 2 and No. 3 or No. 1 and No. 4 or No. 2 and No. 4 increase simultaneously after hybridization.

Hepatitis B caused by the hepatitis B virus (HBV) is a worldwide epidemic and is an infectious disease that causes serious harm to human health. The incidence of hepatitis B in China is high, and the perspectives for prevention and control of HBV are not optimistic. HBV is divided into nine genotypes based on heterology of the complete nucleotide sequence of ≥8% or heterogeneity of the S gene sequence of ≥4%. In China, the predominant genotypes are B and C. The HBV genotype is closely related to viral replication, mutation, the severity of liver disease, chronic process after HBV infection, and antiviral efficacy [1–4]. HBeAg seroconversion appears earlier in genotype B infections than in genotype C infections, and fewer patients with genotype B infections progress to chronic hepatitis, cirrhosis, or liver cancer. Genotype B has a higher response rate to interferon and its mutation rate is low. In short, patients with genotype B HBV infections have better clinical outcomes than patients with genotype C infections. Therefore, accurate and rapid HBV genotyping is of significant importance for the prediction of the risk of progression of HBV infection and selection of the appropriate treatment regimen. Current HBV genotyping methods include sequencing, PCR-RFLP, and gene chip analysis. All of these methods have limitations that restrict their clinical application. A DNA electrochemical biosensor is a device that can convert the presence of target DNA into electrical signals. Compared with the traditional gene detection techniques, biosensors are rapid, sensitive, easy to operate, low in cost, and pollution-free. The research field investigating the use of biosensors has demonstrated the substantial prospects for the application of these methods in both clinical testing and genetic diagnosis of epidemic diseases, genetic diseases, and tumors [5–10]. RuHex does not have non-specific adsorption on the mercaptohexanol surface; therefore, it can be ascertained if RuHex adsorbed on to the electrode surface is bound to the phosphate groups of DNA via electrostatic forces. Therefore, this method has good accuracy and specificity for the detection of target genes [11–15]. This current study employed genotype B and genotype C-specific probes according to thegenome sequences of genotype B and genotype C of Chinese HBV to develop a chronocoulometry method using RuHex as the hybridization indicator for the different genotypes (Figure 1).

## 2. Experimental

### 2.1. Reagents

Tris-(hydroxymethyl) aminomethane was from Sinopharm Chemical Reagent Co., Ltd (Shanghai, China). Hexaamineruthenium III complex (Ru(NH_3_)_6_Cl_3_), ethylenediaminetetraacetic acid (EDTA) and mercaptohexanol (MCH) were purchased from Sigma-Aldrich (St. Louis, MO, USA). Tris-(2-carboxyethyl) phosphine hydrochloride (TCEP) was purchased from Shanghai Sangon Biological Engineering Technology Services Reagents Co., Ltd. (Shanghai, China). The buffer solutions involved in the study were as follows: DNA immobilization buffer was 10 mM tris (hydroxymethyl) aminomethane hydrochloride (tris-HCl), 1.0 mM ethylenediaminetetraacetic acid, 10 mM tris (2-carboxyethyl) phosphine hydrochloride (pH 7.4), and 0.1 M sodium chloride;hybridization buffer was 1 M sodium chloride and 10 mM phosphate buffered saline (pH 7.4); Buffers for measurement were 10 mM tris- (hydroxymethyl)aminomethane hydrochloride (pH 7.4) containing 50 μmol/L Ru(NH_3_)_6_Cl_3_; buffer for electrode washing was 10 mM phosphate buffered saline (pH 7.4); All solutions were prepared with MilliQ water (18 MW cm resistivity) from a Millipore system. The synthetic oligonucleotides (Table 1) were purchased from TaKaRa Biotechnology Co., Ltd (Dalian, China). All the chemicals used were of analytical reagents grade and sterilized and deionized double distilled water (DDW) was used throughout.

### 2.2. Apparatus

All electrochemical measurements were performed on a CHI760D electrochemical workstation (CH Instruments, Inc., Austin, TX, USA). Electrochemical experiments were carried out with a conventional three-electrode system comprising a gold working electrode, a platinum wire auxiliary electrode, and a silver/silver chloride (with saturated potassium chloride) reference electrode. All potentials herein were referred to this reference electrode. Cyclic voltammetry was carried out at a scan rate of 50 mV/s and chronocoulometry at a pulse width of 250 ms. The electrolyte buffer was thoroughly purged with nitrogen before experiments. Viral genomic DNA extraction kit (magnetic beads adsorption, Beijing Sino-Mdgene Technology Co. Ltd, Beijing, China), General DNA purification Kit (Tiangen Ltd., Beijing, China), BioPhotometer UV spectrophotometer (Eppendoff, Brand, Germany), StepOne real-time fluorescence quantitative PCR instrument (Applied Biosystems, Foster, CA,USA), ABI 3130 genetic Analyzer (Applied Biosystems).

#### 2.2.1. Formation of the SAMs at Gold Electrode Surfaces

The bare gold electrode surface (2 mm in diameter) was freshly polished prior to use with 0.3 and 0.05 μm alumina powder, respectively, and then sequentially cleaned ultrasonically in ethanol and water for 3 min. Then electrodes were electrochemically cleaned in fresh 0.5M H_2_SO_4_ solution, then rinsed with a great amount of Milli-Q water. After being dried with nitrogen, electrodes were immediately used for DNA immobilization. The cleaned electrodes were incubated in the immobilization buffer which contained capture probes modified with thiolate at 0.5 μmol/L for 0.5 h at room temperature. After that, the SH-DNA modified electrodes were treated with 1 mM MCH for 1 h to obtain mixed ssDNA/MCH modified electrodes.

#### 2.2.2. Hybridization with Different Target DNAs

The ssDNA/MCH-modified electrode was incubated in the hybridization buffer which contained the different target DNAs at 45 °C for 1h. After that, dsDNA/MCH-modified electrodes were washed with deionized water and immersed in 0.1% sodium dodecyl sulfate phosphate buffer (pH 7.40) to remove unbound oligonucleotides before RuHex accumulation. For DNA hybridization detection with an electrochemical RuHex indicator the probe DNA or hybridized electrodes were firstly immersed into 10 mM tris-HCl solutions (pH 7.4) containing 50 μM RuHex for 3 min and then the cyclic voltammograms and chronocoulometry were recorded online.

## 3. Results

### 3.1. Electrochemical Characterization of the Probes

**Figure 2.**
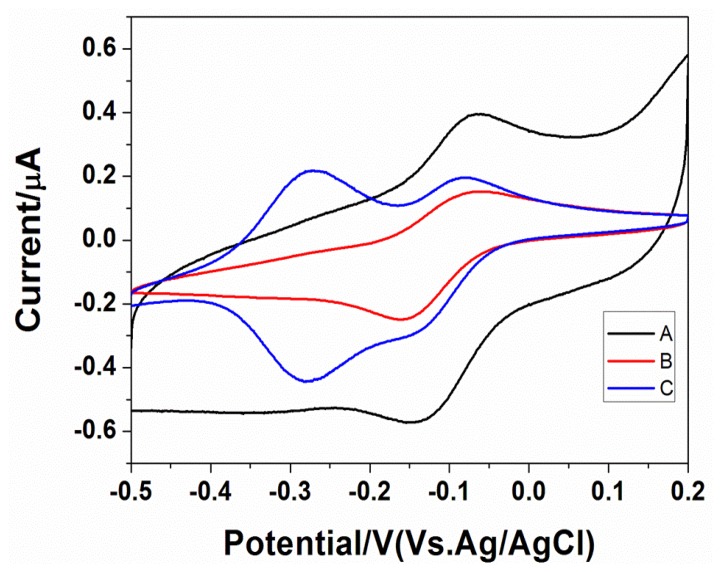
Cyclic voltammograms of electrodes. Bare AuE (A), MCH/AuE (B), DNA/MCH/AuE (C). The electrolyte was 10.0 mM Tris buffer (pH 7.4) containing 50 μM RuHex. Pulse period, 250 ms.

Figure 2 shows the bare gold electrode, mercaptohexanol-modified gold electrodes, probes, and the cyclic voltammogram of the mercaptohexanol-modified gold electrode in 50 μmol/L RuHex solution. The concentration of the probe assembly was 2 μmol/L. It can be seen from the figure that the gold electrode used for assembling the probe had an additional pair of reversible oxidation reduction peaks near −0.3 V when compared to the bare gold electrode and mercaptohexanol-modified gold electrodes. The peak potential difference was less than 39 mV, which was attributed to the characteristic redox peak of ruthenium adsorbed on the electrode surface; this is due to the positively charged ruthenium stably binding to the negatively charged phosphate radicals of the probe DNA through electrostatic interactions. This indicated that the probes were successfully assembled on the electrode surface and that the ruthenium demonstrated non-specific adsorption on to the gold electrodes and mercaptohexanol.

### 3.2. Selection of Probe Assembling Conditions

**Figure 3.**
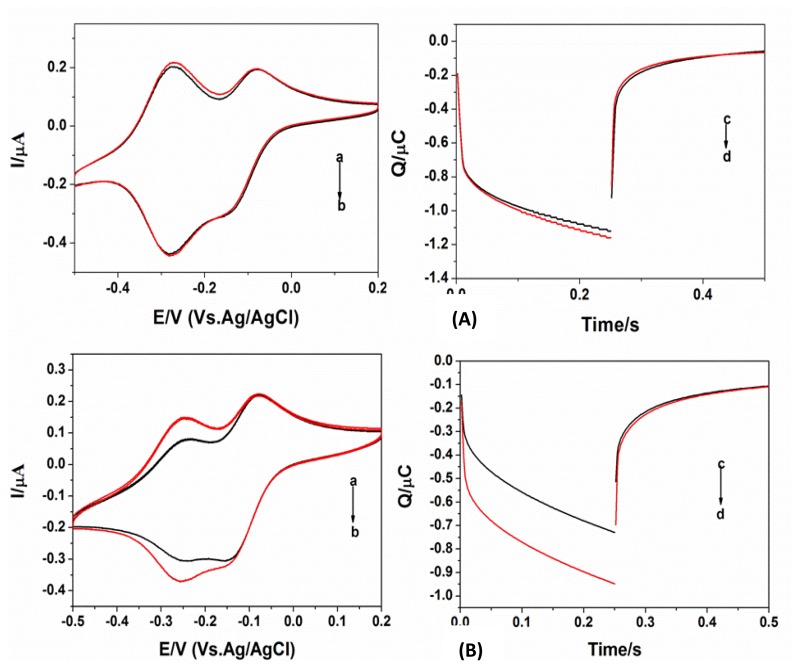
(**A**) Cyclic voltammograms and chronocoulometry curves of the concentration of probe is 2.0 μmol/L. Cyclic voltammograms: dsDNA/RuHex/MCH/AuE(a), ssDNA/RuHex/MCH/AuE (b), chronocoulometry curves ssDNA/RuHex/MCH/ AuE (c), ssDNA /RuHex/MCH/ AuE (d). (**B**) Cyclic voltammograms and chronocoulometry curves of the concentration of probe is 0.5 μmol/L. Cyclic voltammograms: dsDNA/RuHex/MCH/ AuE(a), ssDNA/RuHex/MCH/AuE(b), chronocoulometry curves: ssDNA/RuHex/MCH/ AuE (c), ssDNA /RuHex/MCH / AuE(d).

According to the literature [16–18] when the probes are assembled on the electrode surface at a low density, greater hybridization efficiency can be obtained. Therefore, this study first examined the impact of concentration and duration of probe assembly on hybridization efficiency. Figure 3 indicates the extent of signal differentiation of the high-density and low-density C2 probe assembles. The comparison between Figure 3A and Figure 3B indicated that when the assembly density was high the electric quantity change was small after the hybridization of the probes and target DNA. When the assembly density was low, it can be seen that the adsorption peak at −0.3V was significantly increased after hybridization and the ruthenium diffusion peak at −0.1V did not significantly change, indicating that with the increase in DNA hybridization the amount of ruthenium adsorbed to the electrode surface via electrostatic interactions was substantially increased. Similarly, if the C2 probe was changed to a B1, B2, or C1 probe (with the probes assembled at a concentration of 0.5 μmol/L for 0.5 h) the same results could be obtained. Therefore, we selected these experimental conditions for further testing.

### 3.3. Genotyping of genotype B and C HBV

**Figure 4.**
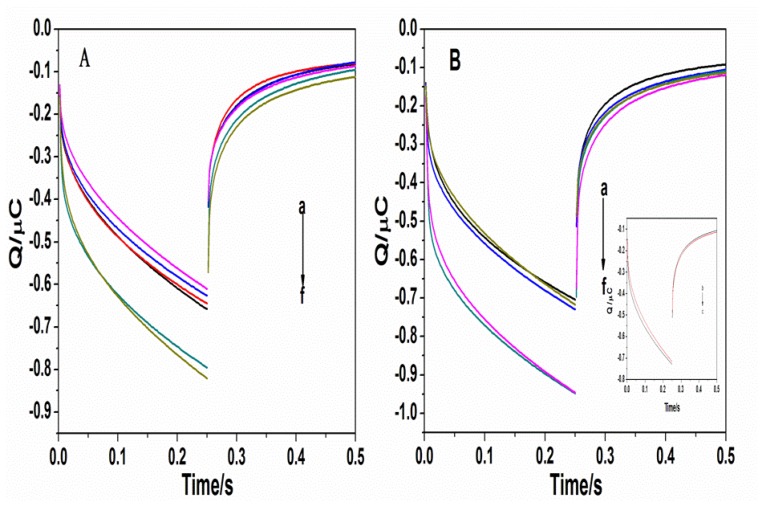
(**A**) Chronocoulometry curves of probe B1 and B2 using 50 μM RuHex as redox indicator. Curves of probe B1 hybridization with noncomplementary sequence (10 nmol/L C1 or C2 target) (a); the blank hybridization (b); curves of probe B2 using 50 μM RuHex as redox indicator: the blank hybridization (c); hybridization with noncomplementary sequence (10 nmol/L C1 or C2 target) (d); complementary target sequence (10 nmol/L B1 target) (e); complementary target sequence (10 nmol/L B1 target) (f). (**B**) Chronocoulometry curves of probe C1 and C2 using 50 μM RuHex as redox indicator. Curves of probe C1 hybridization: the blank hybridization (a); hybridization with noncomplementary sequence (10 nmol/L B1 or B2 target) (b); hybridization with noncomplementary sequence (10 nmol/L B1 or B2 target) added complementary target sequence (10 nmol/L C2 target) (e); chronocoulometry curves of probe C1 using 50 μM RuHex as redox indicator: the blank hybridization (c); hybridization with noncomplementary sequence (10nmol/L B1 or B2 target) (d); hybridization with noncomplementary sequence (10 nmol/L B1 or B2 target); added complementary target sequence (10 nmol/L C1 target) (f).

Figure 4 shows the genotyping map of the No. 1, 2, 3, and 4 electrodes modified with the B1, B2, C1, and C2 probes, respectively

It can be seen from the figure that the No. 1, 2, 3, and 4 electrodes did not have different current signals when they were not bound by the corresponding complementary strands. If two complementary strands for genotype B (B1 and B2 target strands) were added, the No. 1 and 2 electrodes detected a significant difference in electric quantity. Similarly, if two complementary strands for genotype C were added (C1, C2 target strands) during hybridization, the No. 3 and 4 electrodes could detect the corresponding changes. When the target strands were a mix of genotypes B and C, all four electrodes detected the signal differences and they could differentiate genotype B (10%) or C (10%). The correlation of the copy number of oligomers and the electrochemical signal is Q/Copy = 0.05 μC/(10 ^−10^ × 6.022 × 10^23^)/(61 × 1 × 10^9^ × 660) =1.496 × 10 ^−15^C/Copy.

### 3.4. Detection of the HBV Target Sequences at Different Concentrations

**Figure 5.**
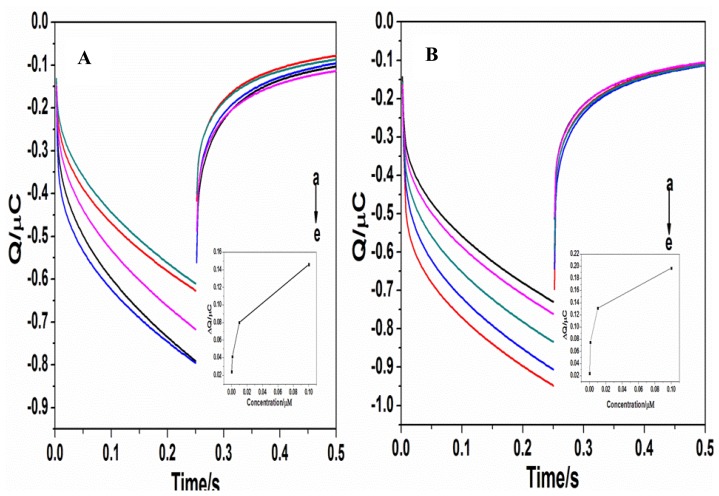
Chronocoulometry curves using 50 μM RuHex as redox indicator for a probe modified electrode after hybridization with different target sequence: 0 nmol/L (a); 0.1 nmol/L (b); 1 nmol/L (c); 10 nmol/L (d); 100 nmol/L (e). The figure (**A**) is for the B2 probe and the figure (**B**) for the C2 probe, respectively. Inset shows the plot of the charge increment of RuHex as a function of the target concentration. Signal was defined as the difference in the redox charge of RuHex after and before hybridization (signal = Q_after_ − Q_before_).

Figure 5 presents the chronocoulometry map of the different concentrations of target sequences detected by the probes

For example, the B2 probe for genotype B was used to detect the B2 target sequence and the C2 probe for genotype C was used to detect the C2 target sequence. It can be seen that within the concentration range (1.0 × 10^−10^ to 1.0 × 10^−7^ mol/L) the electric quantity signal showed an incremental increase with the increase in the concentration of complementary sequences. Therefore, the chronocoulometry method can accurately reflect changes in the concentration of the complementary sequences. The intra-assay CV for B type was 1.93%, 1.81% and 3.80%, inter-assay CV were 4.33%, 2.12%and 4.06%, while the intraassay CV for C type was 2.53%, 4.11%, 2.16%, inter-assay CV were3.55%, 2.16%and 3.10%. The HBV DNA was not detected in any serum samples of HCV, HSV, and HPV and healthy volunteers.

## 4. Discussion

The different HBV genotypes and clinical outcomes are closely related, therefore, HBV genotyping has important clinical significance. Although there are currently many monitoring methods, including the gene chip technique, quantitative PCR, and sequencing, they are not suitable for large-scale clinical detection. Although direct sequencing is the gold standard for virus genotyping, the sensitivity is not high, the operation is time-consuming, and it is difficult to automate the detection process [19]. Gene chips have a high cost and a low sensitivity. In this study, the electrochemical sensing technique was investigated. The cost of this method was greatly reduced because no other materials were needed, and the sensitivity of this method reached 10^−10^ mol/L. Moreover, the technique itself has unparalleled advantages, such as the dual-probe double verification system and rapid high-throughput detection. Using ruthenium as an indicator, this study developed an HBV genotyping technique based on electrochemical DNA sensors by applying the chronocoulometry method as well as dual-probe double verification. Highly sensitive and accurate HBV genotyping was realized and the overall duration of the detection process was significantly reduced to only 2.5 hours. This method is expected to be a substantial improvement in HBV genotyping for clinical practice.

## 5. Conclusions

In general, electrochemical sensing technique for rapid genotyping of HBV shows higher sensitivity compared to conventional PCR/Sanger sequencing by optimization of conditions without requirements of additional cost and equipment. The advantage of significantly reducing the undetected rate by Sanger sequencing will be beneficial to the rational use of drugs or change in treatment in patients with HBV timely.

## Figures and Tables

**Table 1.** Specific genotype probes and relative positions of their target sequences.

**Name**	**Sequence**	**Position**	**Genotype**
B1 capture	3′ -TCTATTAGTAATGAAGGTCTGC-(CH2)6-SH-5′		
B1 target	5′-AGTTAATCATTACTTCCAGACG-3′	nt2717-2738	B
B2 capture	3′ -GACATCTAGAACAAGGGTTCTT-(CH2)6 -SH-5′		
B2 target	5′-CTGTAGATCTTGTTCCCAAGAA-3′	nt2846-2885	B
C1 capture	3′ –ACAAGGCTGATGACGGAGTG-(CH2)6-SH-5′		
C1 target	5′-TGTTCCGACTACTGCCTCAC-3′	nt237-256	C
C2 capture	3′ –CCTGTTTAACCTCCTGTTCTC-(CH2)6-SH- 5′		
C2 target	5′-GGACAAATTGGAGGACAAGAG-3′	nt167-138	C
